# Bile Leak 30 Years Post-cholecystectomy: A Rare Case

**DOI:** 10.7759/cureus.116468

**Published:** 2026-09-18

**Authors:** Rami Ayoub, Seohyun Lee, Konstantinos Baronos, Akshit Goel, Zobia Wadi, Emma Telford, Alice Millard, Shrey Parmar, Htoo Htet Linn, Neil Bhardwaj

**Affiliations:** 1 Department of Hepatobiliary Surgery, Al-Balqa Applied University, Al-Balqa, JOR; 2 Department of Hepatobiliary Surgery, University Hospital Coventry & Warwickshire, Coventry, GBR; 3 Department of Hepatopancreatobiliary Surgery, University Hospitals of Leicester NHS Trust, Leicester, GBR; 4 Department of Medicine, University Hospitals of Leicester NHS Trust, Leicester, GBR; 5 Department of Hepatobiliary Surgery, University Hospitals of Leicester NHS Trust, Leicester, GBR; 6 Department of General Surgery, University Hospitals of Leicester NHS Trust, Leicester, GBR

**Keywords:** persistent bile leak, post-cholecystectomy, post-cholecystectomy bile duct injury, post-cholecystectomy complications, spontaneous bile leak

## Abstract

Bile leaks are a well-recognised complication of cholecystectomy, typically presenting within the first postoperative week. Extremely delayed presentations are exceptionally rare, with only a handful of cases reported years after the index procedure. We report the case of a 60-year-old woman who presented with a four-day history of epigastric pain, fever, and vomiting, 30 years following open cholecystectomy. Imaging revealed a subhepatic fluid collection and a dilated common bile duct suggestive of a biliary leak. She was successfully managed with ultrasound-guided percutaneous drainage and intravenous antibiotics, without the need for endoscopic retrograde cholangiopancreatography, and made an uneventful recovery. This case represents one of the longest documented intervals between cholecystectomy and bile leak presentation, highlighting that biliary causes of intra-abdominal sepsis should not be excluded irrespective of the time elapsed since surgery, and reinforcing the importance of lifelong clinical vigilance following cholecystectomy.

## Introduction

Cholecystectomy is one of the most commonly performed surgical procedures worldwide, with biliary complications representing a significant source of postoperative morbidity. Bile leaks occur in approximately 0.5% of cases following cholecystectomy and are typically identified within the first postoperative week, with a median time from surgery to presentation of five to seven days [1,2]. These leaks most frequently arise from the cystic duct stump, accessory ducts such as the duct of Luschka, or injuries to the common bile duct, and prompt diagnosis is essential to prevent progression to biloma formation, peritonitis, and sepsis [3]. The clinical presentation of bile leaks ranges from subtle biochemical abnormalities to overt peritonitis, and diagnosis often requires a high index of suspicion, particularly when symptoms are atypical or delayed.

While most biliary leaks present acutely within a few days, extremely delayed presentations occurring months to years after are exceedingly rare, with only a handful of cases reported at intervals of four, six, seven, and nine years post-cholecystectomy [4-7]. The adoption of the “critical view of safety,” as described by Strasberg et al., has been instrumental in reducing the risk of biliary injury during laparoscopic cholecystectomy by ensuring unambiguous identification of the cystic duct and artery before division [8,9]. However, the pathophysiological mechanisms underlying such remarkably delayed events remain incompletely understood, and the diagnostic workup in these patients presents unique challenges distinct from those of acute postoperative leaks.

We report the case of a 60-year-old woman presenting with a bile leak 30 years following cholecystectomy, which is one of the longest documented intervals in the literature.

## Case presentation

A 60-year-old Polish woman presented to the emergency department with a four-day history of epigastric abdominal pain radiating to the right iliac fossa and to the back. The pain was persistent and sharp in character, associated with fever, nausea, and vomiting of solid brown water contents, without a bowel movement for four days. Past medical history included osteoarthritis, hypertension, chronic plaque psoriasis, gout, and mechanical lower back pain requiring multiple sacroiliac joint steroid injections. She had a right middle cerebral artery infarct four years ago with residual left-sided sensory impairment. Surgical history included a caesarean section and an open cholecystectomy performed over 30 years previously. She was an active smoker and denied alcohol use.

On examination, she was systemically unwell with oxygen saturation of 95% on room air, respiratory rate of 21 breaths per minute, heart rate of 116 beats per minute, blood pressure of 119/80 mmHg, and temperature of 38.5°C. Abdominal examination revealed marked tenderness and guarding in the right iliac fossa. Healed surgical scars were noted, including a right upper paramedian incision consistent with prior cholecystectomy and a Pfannenstiel incision.

Initial blood tests confirmed ongoing sepsis with a raised white cell count of 17.0 × 10⁹/L (neutrophils: 13.48 × 10⁹/L) and C-reactive protein of 270 mg/L. Liver function tests were deranged; bilirubin was 31 µmol/L and alkaline phosphatase was 203 IU/L, while alanine aminotransferase was 39 IU/L and albumin was 42 g/L. Amylase, renal function, and coagulation profile were within normal limits. Urinalysis was positive for leukocytes, nitrites, protein, blood, and bilirubin; urine culture subsequently grew *Escherichia coli* (KESC group).

Computed tomography (CT) with contrast of the abdomen and pelvis demonstrated a large volume of subhepatic free fluid within the gallbladder fossa extending into the right flank and retroperitoneum. The common bile duct was dilated to 1.2 cm with abrupt distal tapering, accompanied by mild intrahepatic duct dilatation. The appendix appeared normal, and there was no evidence of pneumoperitoneum. Incidental findings included a duodenal diverticulum (D3), a small non-obstructing right renal calculus, and a small right pleural effusion with bibasal atelectasis (Figure 1).

**Figure 1:**
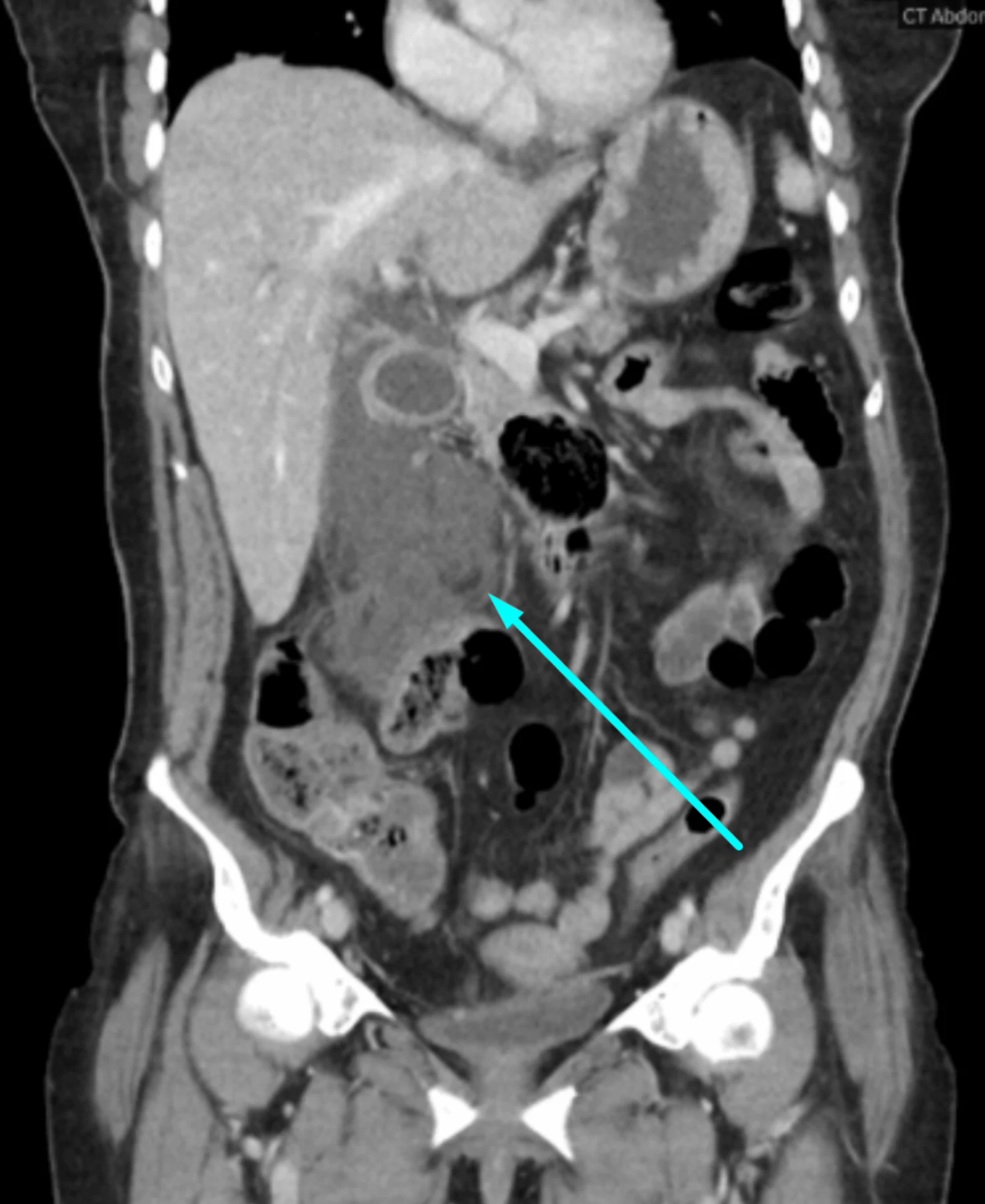
Contrast-enhanced coronal CT of the abdomen and pelvis demonstrating a large subhepatic fluid collection extending from the gallbladder fossa into the right flank and retroperitoneum (arrow).

Magnetic resonance cholangiopancreatography (MRCP) was then requested to further evaluate the bile leak and revealed an extensive irregular subhepatic fluid collection extending inferiorly from the gallbladder fossa. The distribution was suggestive of a cystic duct stump leak, although no definitive leak was visualised, partly due to motion artefact. The biliary tree appeared prominent without evidence of intraluminal filling defects (Figure 2).

**Figure 2:**
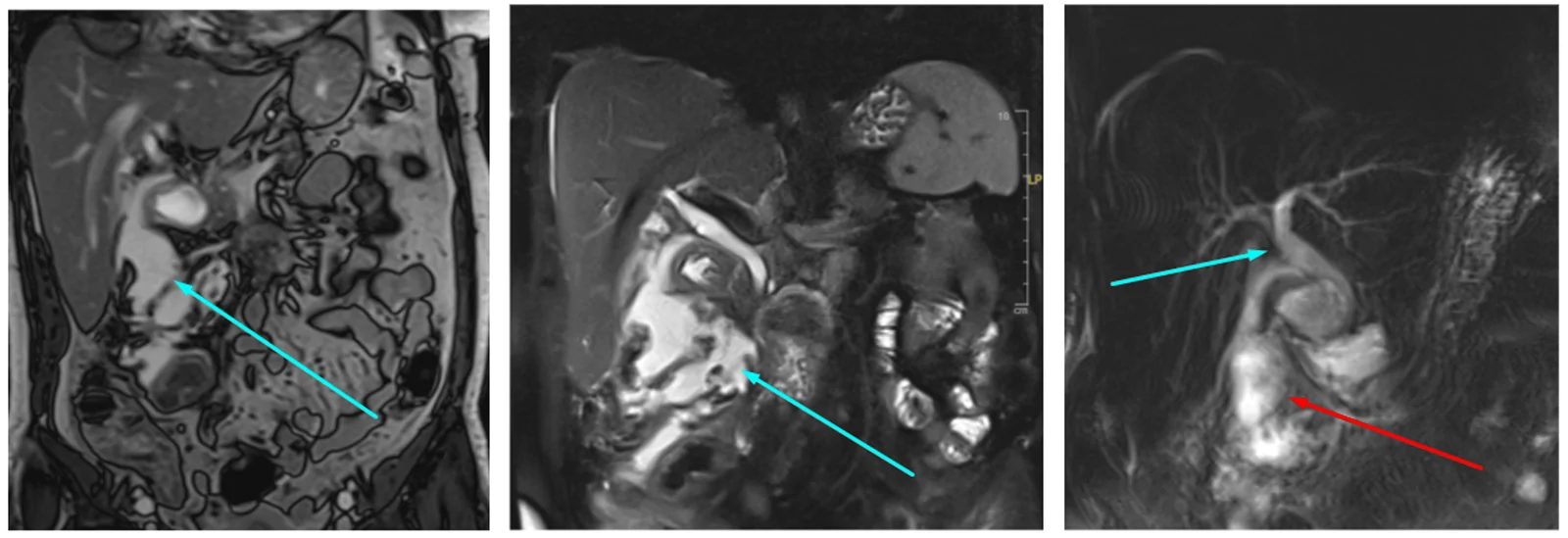
Magnetic resonance cholangiopancreatography (MRCP) demonstrating an extensive irregular subhepatic fluid collection extending inferiorly from the gallbladder fossa, suspicious for a cystic duct stump leak. (A, B) Coronal images showing the subhepatic fluid collection (arrow). (C) MRCP image showing the cystic duct stump (aqua arrow) in relation to the adjacent fluid collection (red arrow).

She was commenced on intravenous antibiotics (meropenem), patient-controlled analgesia with intravenous morphine, regular paracetamol, and intravenous fluids. Ultrasound-guided percutaneous drainage of the subhepatic collection was performed, with insertion of a 10 Fr drain yielding dark bile fluid. Following drainage and continued antibiotic therapy, her inflammatory markers improved. She was discharged on a further five-day course of oral co-amoxiclav.

At the one-week outpatient review, the drain remained in situ with approximately 100 mL of dark bile fluid output, and blood tests had normalised. At two weeks, there was no further output, and the drain was flushed. By three weeks post-discharge, the drain was removed with no recurrence of symptoms.

## Discussion

This case represents one of the longest reported intervals between cholecystectomy and presentation of a bile leak, with the vast majority presenting within the first postoperative week. In large endoscopic series, the median time from surgery to endoscopic retrograde cholangiopancreatography (ERCP) for bile leak is typically five to seven days [1,10]. Even rarer are bile leaks presenting months to years later, with only a handful of cases reported at four, six, seven, and nine years post-cholecystectomy [4-7]. Our case, presented 30 years after an open cholecystectomy, represents one of the longest documented intervals and demonstrates just how delayed a biliary complication can present.

The mechanisms underlying such an extremely delayed leak remain uncertain. One potential explanation is a minor iatrogenic and unrecognised damage to an aberrant bile duct, such as the right posterior sectoral duct or the subvesical duct of Luschka, that remained clinically silent for years [6,7]. Biliary anomalies are present in up to 40% of the population, and injuries to these ducts are not as common and are likely underreported, as they remain clinically silent and undetected due to progressive atrophy of the affected areas [2,6].

Another possibility is a chronic encapsulated biloma which slowly enlarged and became superinfected, which would explain the lack of a clear communication site on MRCP [6,7]. Delayed recurrent leakage secondary to an unrecognised right posterior sectoral duct injury has previously been described several years following cholecystectomy [6]. In our patient, imaging demonstrated a dilated common bile duct (1.2 cm) with abrupt distal tapering and mild intrahepatic duct dilatation, without any obstructing calculus identified. Increased intraductal pressure may therefore have precipitated leakage from a pre-existing vulnerable site related to the original surgery.

Diagnostically, this case illustrates the limitations of non-invasive imaging. While CT and ultrasound are sensitive for detecting fluid collections, they cannot reliably distinguish bile from other fluids. MRCP provides detailed anatomical information, and in this case, the subhepatic collection was suggestive of a bile leak or collection. ERCP is particularly valuable as it provides both diagnostic localisation and therapeutic intervention [11]. In our patient, ERCP was not performed. Therefore, despite clinical and radiological evidence strongly supporting a biliary source, the precise anatomical site of leakage remains uncertain.

Management strategies for postoperative bile leaks are well-established. ERCP with biliary stenting, with or without sphincterotomy, remains the gold standard, achieving clinical success in approximately 90% of cases in many published series [9,10]. ERCP with stenting reduces the transpapillary pressure gradient between the bile duct and duodenum, allowing the damaged duct to heal while also physically blocking the defect [11]. However, in our patient, the decision to proceed with percutaneous drainage and antibiotics, without ERCP, successfully led to resolution. This outcome suggests that the leak was likely a low-flow defect or already closing spontaneously, and demonstrates that drainage alone may be sufficient in selected stable patients, with ERCP reserved for persistent or recurrent leakage.

This case has other limitations. First, the exact site and nature of the bile leak were not confirmed by imaging or endoscopic visualisation. Second, the 30-year interval precludes verification of the original operation details, so the exact mechanism of the leak remains uncertain. Third, bile cultures were not obtained from the drained fluid, so the microbiological aetiology of the sepsis is inferred.

## Conclusions

This case highlights that in any patient with a background of cholecystectomy, no matter how delayed the presentation, a biliary cause of intra-abdominal sepsis should not be excluded. Ultimately, this case demonstrates that a past history of cholecystectomy remains a critical diagnostic consideration when evaluating any new-onset, unexplained subhepatic collection or intra-abdominal sepsis, reinforcing the need for long-term clinical awareness.
